# Neuropathology of incidental Lewy body & prodromal Parkinson’s disease

**DOI:** 10.1186/s13024-023-00622-7

**Published:** 2023-05-12

**Authors:** Thomas Koeglsperger, Svenja-Lotta Rumpf, Patricia Schließer, Felix L. Struebing, Matthias Brendel, Johannes Levin, Claudia Trenkwalder, Günter U. Höglinger, Jochen Herms

**Affiliations:** 1 Department of Neurology, LMU University Hospital, LMU Munich, Munich, Germany; 2 Department of Translational Brain Research, DZNE-German Center for Neurodegenerative Diseases, 81377 Munich, Germany; 3 Centre for Neuropathology and Prion Research, LMU Munich, Munich, Germany; 4 Department of Nuclear Medicine, LMU University Hospital, LMU Munich, Munich, Germany; 5 Munich Cluster for Systems Neurology (SyNergy), 81377 Munich, Germany; 6 Clinical Study Unit, DZNE - German Center for Neurodegenerative Diseases, 81377 Munich, Germany; 7 Paracelsus-Elena Klinik, Kassel, Germany; 8 Department of Neurosurgery, University Medical Center Goettingen, Goettingen, Germany; 9 Department of Neurology, Medizinische Hochschule Hannover (MHH), Hannover, Germany

**Keywords:** Prodromal Parkinson’s disease (PD), Incidental Lewy body disease (iLBD), Lewy body α-Synuclein, Enteric nervous system (ENS), REM-sleep behaviour disorder (RBD)

## Abstract

**Background:**

Parkinson’s disease (PD) is a progressive neurodegenerative disorder associated with a loss of dopaminergic (DA) neurons. Despite symptomatic therapies, there is currently no disease-modifying treatment to halt neuronal loss in PD. A major hurdle for developing and testing such curative therapies results from the fact that most DA neurons are already lost at the time of the clinical diagnosis, rendering them inaccessible to therapy. Understanding the early pathological changes that precede Lewy body pathology (LBP) and cell loss in PD will likely support the identification of novel diagnostic and therapeutic strategies and help to differentiate LBP-dependent and -independent alterations. Several previous studies identified such specific molecular and cellular changes that occur prior to the appearance of Lewy bodies (LBs) in DA neurons, but a concise map of such early disease events is currently missing.

**Methods:**

Here, we conducted a literature review to identify and discuss the results of previous studies that investigated cases with incidental Lewy body disease (iLBD), a presumed pathological precursor of PD.

**Results:**

Collectively, our review demonstrates numerous cellular and molecular neuropathological changes occurring prior to the appearance of LBs in DA neurons.

**Conclusions:**

Our review provides the reader with a summary of early pathological events in PD that may support the identification of novel therapeutic and diagnostic targets and aid to the development of disease-modifying strategies in PD.

## Pathology progression and staging in PD

### The traditional concept of pathology progression in PD

**Fig. 1:**
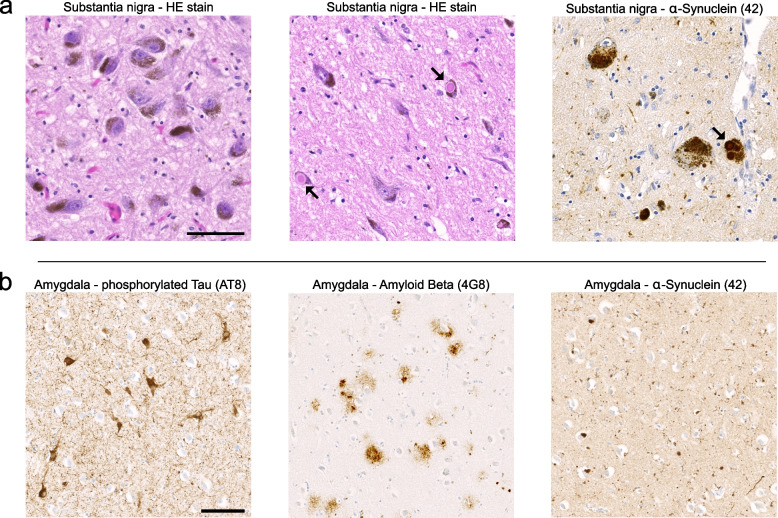
Neuropathological hallmarks of Parkinson’s disease and associated co-pathologies. **a** The neuropathological hallmarks of PD are shown in the three sub-panels. From left to right: a normal substantia nigra without any PD features; substantia nigra tissue from a PD patient with Lewy bodies (arrows) and pigmented/dopaminergic neuron loss; an immunohistochemical stain against αSyn (clone 42) from the same PD patient demonstrating intraneuronal Lewy bodies and Lewy neurites. Note that melanin pigment and DAB stain are not easy to distinguish. Scale bar = 50 μm. **b** Immunohistochemically stained sections of the amygdala from a patient suffering from mixed-type dementia that presented clinically as PD. The amygdala is a region commonly affected by co-pathologies. From left to right: An antibody against phosphorylated tau protein (clone AT8) demonstrates neurofibrillary tangles; immunohistochemistry against amyloid beta (clone 4G8) highlights diffuse and cored amyloid beta plaques; the clone 42 against αSyn marks Lewy bodies, Lewy neurites and a few axonal spheroids. Scale bar = 100 μm

**Fig. 2:**
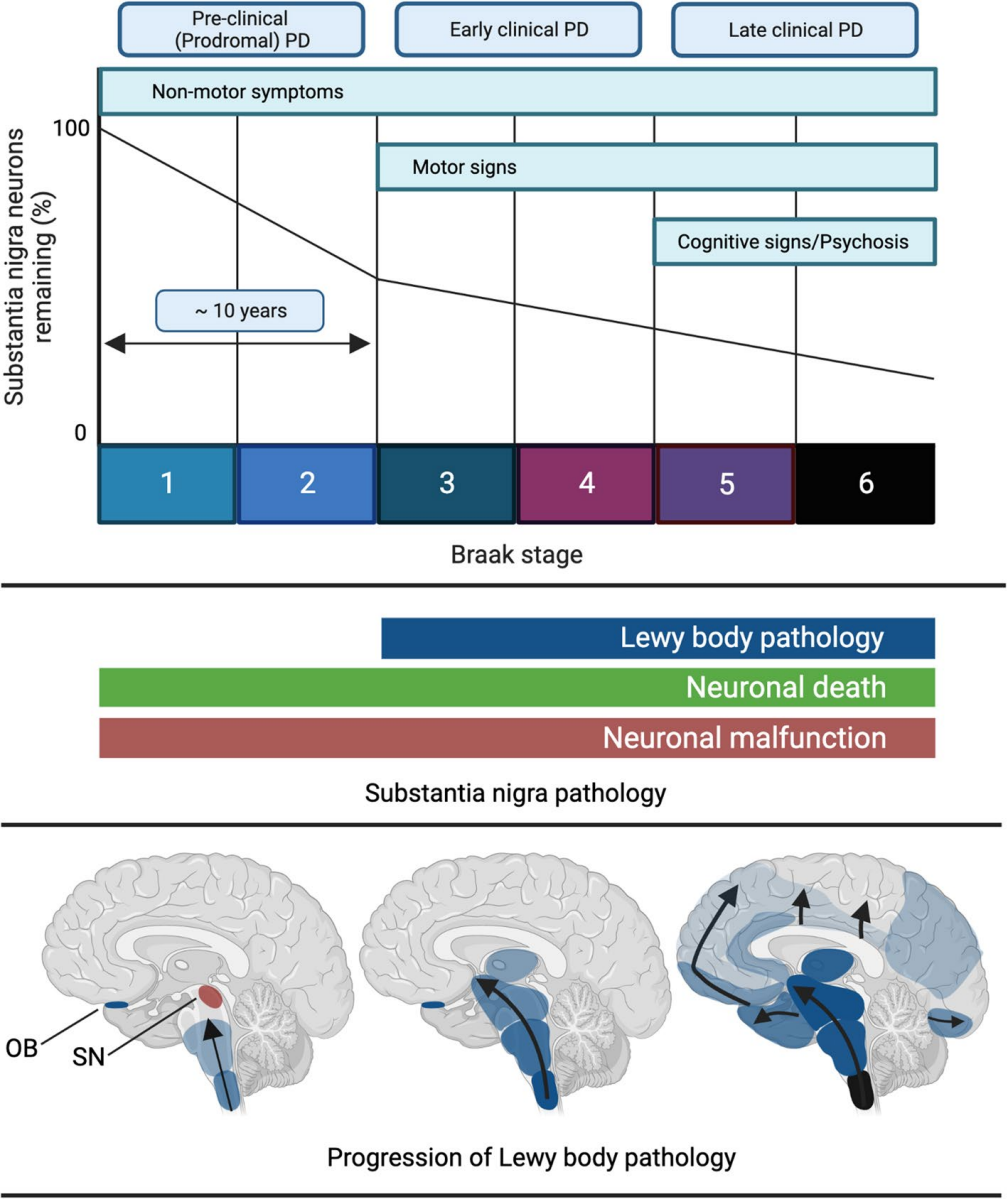
Schematic illustrating disease progression from the pre-clinical phase of PD to the late clinical phase, including the associated clinical symptoms, Substantia nigra pathology and spread of Lewy body pathology according to Braak’s staging model. Note the presence of neuronal dysfunction in the SNc prior to the appearance of Lewy bodies in this area. *Created with Biorender.com*

Parkinson’s disease (PD) is the most common neurodegenerative movement disorder and is characterised by the progressive development of bradykinesia, muscular rigidity, rest tremor, and postural instability [1]. The cardinal motor features result from the progressive loss of dopaminergic (DA) neurons in the substantia nigra pars compacta (SNc) [2]. A neuropathological hallmark of PD are neuronal protein aggregates termed Lewy bodies (LBs) (Fig. 1a) [3]. LBs are composed of vesicular membrane structures and dysmorphic organelles in conjunction with protein aggregates containing alpha-Synuclein as the main component (αSyn) [4, 5]. Gene multiplications and missense mutations in *SNCA,* the gene coding for αSyn, are causative for familial forms of PD, which account for 10–15% of cases [6–9]. In addition, genome-wide association studies linked common variants at the *SNCA* locus to sporadic PD, thus further supporting an important pathogenic role of α-Syn in PD [10]. Postmortem studies suggested that the gradual appearance of LBs correlates with disease progression in PD [11]. Based on the gradual appearance of LBs, Braak et al*.* developed a neuropathological staging scheme for PD. The authors proposed that PD primarily starts in the olfactory bulb and the autonomic enteric nervous system (ENS), with a caudo-rostral (retrograde) spread of Lewy body pathology (LBP) over time, ultimately reaching the SNc where it is suspected to initiate the demise of DA neurons (Fig. 2) [12–14]. LBs are therefore considered to be a marker for disease progression, while neuronal loss represents a well-established neuropathological correlate of clinical PD (cPD) symptoms [3, 15, 16].

Braak’s staging is divided into six different stages that reflect the progression of LBP from the dorsal motor nucleus of the vagus nerve (DMV) (Braak stage 1) to the locus coeruleus (LC) (Braak stage 2), SNc and amygdala (Braak stage 3) and ultimately reaching cortical areas (Braak stage 4–6) [17, 18]. The Braak stages correspond to the type and degree of clinical symptoms associated with disease progression. Early stages are characterised by non-motor symptoms, while typical PD motor signs are thought to appear once the SNc is affected at Braak stages ≥ 3, and cognitive symptoms arise only as LBP reaches the cortex in Braak stages 5 and 6 (Fig. 2). Braak’s model proposes that LBP gradually appears in defined anatomical structures during disease progression [3, 19]. In line with the idea of a prion-like LBP ‘spreading’ mechanism, fetal DA neurons transplanted into the PD SNc exhibited proteinaceous inclusions that resembled LBs [20, 21]. This result was interpreted as a ‘spread’ of LBP from the host to the graft. In mice, synthetic pre-formed αSyn fibrils propagate from the site of stereotaxic injection to synaptically connected, neighboring structures, thereby creating a Lewy-like pathology [22–25]. Similarly, proteins extracted from human brains with LBP and injected into the striatum of monkeys can also propagate to neighboring structures, illustrating LBP's propensity to spread from its origin [26, 27]. In summary, previous results support a model where LBP gradually builds up in clearly defined brain regions, leading to neuronal death. This process occurs according to a well-defined pattern, resulting in some brain areas remaining unaffected until the final stages of PD, whereas others are devastated by degeneration early on.

### Controversies and limitations of the braak staging scheme

The co-incidence of DA neuron loss and LBs initially primed the conclusion that these intraneuronal inclusions—in combination with cell death—were responsible for the disease. However, a considerable body of research appears to give rise to concerns regarding the overall significance of LBP. For instance, Gibb et al. reported an age-dependent increase in the prevalence of LBs from 3.8% to 12.8% between the sixth and ninth decades of age. This amount exceeds the prevalence of PD by about three- to six-fold [28, 29]. In line, previous reports found that a significant proportion of neuropathologically confirmed LBD cases never exhibited clinical symptoms [30]. In addition, more recent research demonstrated that cell death and LBP do not entirely correlate in the affected brain regions. For instance, even in the absence of LBP, there is considerable neuronal death in the supraoptic nucleus in PD. By contrast, there is no discernible neuron loss in the neighboring, LB-rich tuberomammillary nucleus of the hypothalamus [31]. Furthermore, in patients who do not exhibit dementia during disease progression, the only cortical region that shows substantial neuronal loss is the pre-supplementary motor cortex, where small intra-telencephalic pyramidal neurons degenerate in the absence of LBP [32, 33]. These results cast doubt on the concept that cell death is a consequence of LBP in PD.

Conflicting with other reports, which claim that neurons are primarily lost in brain regions with LBP [34–36], a study from Iacono et al. found no significant correlation between neuronal loss and LBs in the PD brain [37]. Another line of evidence for an LBP-independent pathological process comes from PD cases carrying genetic mutations where LBP distribution is distinct from that of idiopathic PD [38]. For instance, only a part of PD patients with a G2019S mutation in the *LRRK2* gene exhibit LBP and most patients with other *LRRK2* mutations do not even show LBP at all [39] despite substantial SNc DA neuronal degeneration. Likewise, PD cases with *PARK2* mutations have only sparsely distributed LBP with a pattern distinct from that found in idiopathic PD cases [40], although these cases may be phenotypic variations. Finally, conflicting evidence comes from neuropathological and histological studies. Because the number of LBs in patients with mild to moderate SNc neuron loss was higher than in patients with severe neuronal depletion, LB-containing neurons have been initially assumed to be the dying neurons [41]. However, Tompkins and Hill demonstrated that the presence of LBs does not predict a higher degree of cell death compared to the general population of SNc neurons and that most neurons that undergo cell death do not even contain LBs [42].

Moreover, whether SNc neurons contain LBs or not, they are similarly affected by morphological dendritic abnormalities or biochemical changes, indicating that DA neurons, in general, are involved in a yet-to-be-defined disease process [43–46]. These results imply that region-specific environmental changes may prime these DA neurons to preferentially degenerate in PD. Consequently, attempts to correlate the density of either cortical or brain stem LBs with the progression and severity of clinical PD symptoms were unsuccessful [47–50]. Along these lines, in a certain percentage of PD patients who developed dementia, no LBs could be detected in cortical areas or other areas outside the brain stem [51, 52], and these cases may suffer from concomitant amyloid beta (Aβ) pathology. Conversely, the simultaneous presence of Lewy body pathology and Alzheimer's Disease (AD)-related changes, such as hyperphosphorylated tau protein or Aβ, can also be observed (Fig. 1b). Conversely, LBP, which is typically found in the amygdala, is frequently detected in AD cases [53].

Collectively, these findings indicate that the pathophysiology of neurodegeneration and cell death can hardly be explained by LBs or LBP-related cell death alone. Alternative views are that LB formation is a process for detoxification of pathological αSyn aggregates located at a harmful site in the neuron, such as the presynapse [54]. Studies investigating the ultrastructure of LBs indicated that they are formed in an aggresome-related process and support the notion that LBs are a way of containment of protein aggregates and degraded organelles [5, 55]. By using correlative light and electron microscopy and tomography on postmortem human brain tissue from PD brains, the study done by Shahmoradian et al*.* found a crowded environment of membranes in LBs, including vesicular structures and dysmorphic organelles [5]. Crowding of organellar components was confirmed by stimulated emission depletion (STED)-based super-resolution microscopy, and a high lipid content within LBs was corroborated by confocal imaging, Fourier-transform coherent anti-Stokes Raman scattering infrared imaging and lipidomics. The latter report suggests that lipid membrane fragments and distorted organelles, together with a non-fibrillar form of αSyn, are the main structural components of LBs and that they do not contain fibrillar αSyn aggregates. Although a matter of current debate [56], these results thus challenge the pivotal role of αSyn and point towards cellular and molecular changes that may occur independent from the formation of LBP. This view is supported by studies implicating mutations in *PINK1* [57] and lysosomal genes in PD, such as *GBA* [58–61]. Conversely, out of 2000 PD cases, only 0.05% had mutations in the *SNCA* gene, creating uncertainty about how αSyn accumulates in the other 99.95% of cases and whether the protein has a causative role in PD [62]. In summary these results thus cast doubt on the significance of LBP as the sole disease-causing factor in PD and alternative models are required to explain such apparent discrepancies.

### Investigating pathological precursors to determine the significance of LBP in PD

A small number of conceptual approaches attempted to dissect LBP-dependent from LBP-independent events in the PD brain. Besides comparing LBP-affected and unaffected neurons, one strategy has been the examination of pathological precursors of LBP. In addition to cPD, there are a few cases that exhibit brain stem-restricted LBP in the absence of the characteristic clinical PD symptoms and these cases are referred to as incidental Lewy body disease (iLBD) [14, 63, 64]. iLBD occurs in 10–15% of people over 60 years of age [65] and is assumed to represent a pathological precursor in PD [66–69]. Whether these cases constitute a neuropathological PD precursor has been a matter of controversy for some years, as there is no proof that these cases would have progressed to PD if they had survived longer or instead simply recapitulated features of normal brain ageing [70]. However, the study of consecutive cases in large case series and the recognition of several intermediate degrees of involvement of the brain stem, limbic structures and, eventually, the cerebral cortex supports the argument for iLBD as a pathological precursor stage of PD. Since SNc neurons are, by definition, still spared from LBs in iLBD, investigating SNc neurons in these cases may provide insight into the cellular and molecular changes occurring at this critical site in the absence of LBP [71], thus allowing to distinguish LBP-dependent from LBP-independent changes. Moreover, investigating SNc neurons in iLBD may support the understanding of early pathological events occurring prior to the appearance of LBP. Because therapeutic approaches that delay or slow down disease progression in PD are likely to be more effective prior to neuronal cell death, the examination of SNc neuronal changes in iLBD cases may aid to the identification of novel therapeutic targets and, ultimately, to the development of early-acting, potentially disease-modifying interventions. Based on these two reasons, we believe that investigating SNc neuronal changes in iLBD warrants further research. Here, we will review the evidence available from earlier studies that examined the molecular and cellular changes in the iLBD SNc to create a concise map of early neuropathological events during PD disease progression and to nurture prospective research in this direction.

## Neuropathology of incidental LBD (iLBD)

### Structural changes in iLBD

Neuropathological studies suggest that by the time a patient is diagnosed with PD based on clinical motor symptoms, a significant proportion of DA neurons is already lost (Fig. 2) [72] and within four years of diagnosis, DA terminals in the dorsal putamen almost entirely disappeared [73]. Therefore, estimates propose that at least cell loss in the brain commences 5 to 10 years prior to the clinical diagnosis [74, 75]. Indeed, several previous studies investigating neuropathological changes in iLBD demonstrated structural changes in SNc DA neurons in the absence of LBs (Table 1, Fig. 2). In accord with an early neuronal malfunction, previous studies demonstrated a substantial (10–20%) loss of SNc DA neurons and impaired nigrostriatal integrity at Braak stages 1 and 2 [76–78]. Likewise, Dijkstra et al. found a 20% decrease in SNc neuronal cell density in iLBD compared with controls [3]. More recently, Iakono et al. demonstrated a marked nigral neuronal loss in PD and iLBD compared to control cases [37]. Milber et al. have shown that neuronal dysfunction and cell loss may precede LBP in the SNc because prior to the appearance of LBs, these processes were observed in the SNc in iLBD at comparable levels to those of higher Braak stages [79]. In accord with a functionally relevant disease process occurring prior to LBP, PD motor symptoms have even been reported at stage 2 of Braak [80]. All these results are also further supported by case reports [81]. These findings illustrate the need for further investigation at these early stages to account for the neuronal loss before the onset of LBP in this area.

**Table 1:** Table summarizing structural and functional changes in the iLBD midbrain

Early changes in the iLBD midbrain	Ref
**Structural**	
↓ TH neurons in SNc	[3, 76, 77, 77, 78, 78, 79]
↓ TH-positive terminals in the striatum	[73]
**Neurochemical**	
↓ Striatal TH	[71, 82]
↑ Oxidative damage in the SNc	[83]
↑ Neuroketals	[83, 84]
Changes to iron metabolism	[85]
**Autophagy**	
↓ Autophagy-associated SNARE molecules (SNAP29)	[86]
Association of p62 with αSyn inclusions	[87]
**Immunological**	
↑ TLR-2-positive microglia in the SNc	[88]
↑ CD68-positive microglia/macrophages in the SNc	[89]
↑ PAR-2-positive microglia in the anterior cingulate cortex (ACC)	[90]
↑ MCM2-positiv cells in the hippocampus (HC)	[88]
Changes to expression of inflammatory and trophic molecules in the SNc and striatum	[91]
↑ CD8-positive T-cells in the SNc	[92]
↑ Angiogenesis marker a_v_β3	[89]
**Synaptic**	
↓ DA synaptic terminals	[82, 93, 94]
↑ αSyn aggregates at presynaptic terminals	[95]
**Gene Expression**	
Deregulation of genetic networks associated with axon de-/regeneration, immune response, and endocytosis pathways in the SNc	[96]
↑ mtDNA mutation in the SNc	[97]
Alteration of oligodendrocytes and their precursors	[98, 99]
Transcriptomic alterations in the cortex	[100]
**Proteomic**	
Aberrant ApoE and low-density lipoprotein receptor-related protein 1 in SNc DA neurons	[101]
Changes in neuropeptides and glutathione levels	[102, 103]
↑ Sialylation	[104]
↓ Sulfation	[105, 106]
**Neuronal function and excitability**	
↓ FMRP	[107]
**Peripheral changes**	
↑ p-αSyn in the vagus nerve	[108]
↓ Frequency bowel movements	[109]
LBs in the submandibular glands, cervical superior ganglia, cervical sympathetic trunk and vagal nerves	[110]
↓ TH in epi- and myocardial sympathetic nerve fibers	[111]

### General neurochemical changes in iLBD

In addition to these structural changes (i.e., cell loss), investigating iLBD brains revealed certain neurochemical alterations. For instance, Dickson et al. found that tyrosine hydroxylase (TH) immunoreactivity in the striatum was decreased in iLBD compared to normal controls, but not to the same extent as in PD [71]. TH is an enzyme critical for DA production, and its decrease in iLBD indicates a nigrostriatal system that is already impaired at this early stage. Using quantitative ELISA, Beach et al. demonstrated that striatal TH showed a 49.8% reduction in iLBD cases compared to control cases [82]. Together with the morphological studies described above, these reports suggest an early neurochemical alteration of SNc DA neurons prior to the appearance of LBs. Other research groups have provided additional findings on the early pathological changes in PD, including neurochemical or metabolic changes. For instance, early oxidative damage was found in the SNc in iLBD, where nitrated αSyn is already present in small granules in DA neurons before the appearance of LBs [83]. The authors thus concluded that oxidative damage is an early event in PD and may precede the formation of LBs.

In the context of the Renin-angiotensin system (RAS), it is intriguing to note that although this hormonal system is traditionally associated with regulating blood pressure, there is significant interplay with the DA system [112, 113]. Studies have demonstrated that angiotensin blockers can exert a neuroprotective effect on midbrain DA neurons both in vivo and in vitro by reducing oxidative stress, thereby indicating their potential as a therapeutic option. For instance, a retrospective study focusing on patients receiving angiotensin blockers as treatment for hypertension showed a reduced risk of developing PD [114]. Similarly, an analysis of data from ischemic heart disease patients revealed that those prescribed with angiotensin II inhibitors—which have the capacity to cross the blood–brain barrier—had a lower risk of developing PD [115]. These findings underscore the potential of these compounds to counteract the early oxidative damage that primes DA neurons for degeneration, thereby presenting a promising strategy for reducing PD risk.

Further biochemical studies have shown increased levels of neuroketals in the SNc in post mortem tissue from Braak stages 1 and 2, supporting the notion that oxidative damage to specific lipids in the SNc occurs at very early stages of PD and prior to the appearance of LBP [83, 84]. In line, recent observations have shown the concentration of L-ferritin in the SNc to be lower in iLBD (and PD) compared with controls, whereas H-ferritin in PD was found to be higher than in iLBD and controls. This illustrates the subtle abnormalities in iron metabolism in the SNc at the early stages of PD [85]. Summarising these results, neurochemical changes occurring prior to LBP may contribute to the increased propensity of SNc DA neurons to degenerate.

### Changes in autophagy

In line with these neurochemical changes, a report demonstrated p62 immunoreactivity in association with abnormal αSyn inclusions at the early stages of LBP, thus suggesting premature alterations to autophagic pathways in these cases [87]. Tang et al. recently investigated autophagy-associated SNARE molecules in post mortem brain tissue from LBD cases and found a stage-dependent decline of the v-SNARE SNAP29 – a member of the SNARE complex mediating autophagolysosome fusion – as early as in Braak stage 1 (Table 1) [86]. Additional experiments in cultured dopaminergic neurons demonstrated αSyn overexpression to reduce autophagy turnover by compromising the fusion of autophagosomes with lysosomes, thus leading to a decrease in the formation of autophagolysosomes. Mechanistically, αSyn interacted with and decreased the abundance of SNAP29 in vitro. Furthermore, SNAP29 knockdown mimicked the effect of αSyn on autophagy, whereas SNAP29 co-expression reversed the αSyn-induced changes on autophagy turnover and ameliorated DA neuronal cell death. These results thus demonstrated a previously unknown capacity of αSyn to affect intracellular autophagy-associated SNARE proteins and, consequently, reduce autophagolysosome fusion. Most notably, this effect may be evident before the presence of LBs in the SNc. Whereas SNAP29 loss has been identified in SNc neurons in iLBD, the cell culture work is derived from αSyn over-expression, thus making it difficult to compare the two results. One possible explanation is that oligomeric αSyn, not yet aggregated into LBs, may cause such cellular changes during early pathology, although specific αSyn-species remain to be identified.

Oligomers, which are small aggregates of misfolded proteins, are believed by some to be a key contributor to the neurodegenerative processes that occur in PD [116]. These oligomers are thought to be more toxic than other forms of αSyn, such as monomers or fibrils, and have been shown to impair the function of neurons in cell culture and animal models of PD. Furthermore, recent research has indicated that αSyn oligomers can spread from cell to cell in a prion-like manner, propagating the disease throughout the brain [117]. This has led to the hypothesis that targeting αSyn oligomers could be a promising therapeutic strategy for PD. Whereas LBP is visible with histologic methods αSyn oligomers remain undetectable with routine approaches but may be an important contributor of early pathological changes. Detecting αSyn oligomers requires special techniques, and their distribution and association with clinical features are important research objectives. Recent advances in detecting αSyn oligomers, such as using proximity ligation assay (PLA) [118] or oligomer-specific antibodies [119] may support investigating such early pathological changes in PD.

### Immunological changes in iLBD

Following clinical reports [120], a recent immunohistochemical study assessing the abundance of the inflammation-associated Toll-like-Receptor 2 (TLR-2) showed increased numbers of TLR-2-positive microglia in the iLBD SNc compared to PD [88], suggesting inflammatory changes occur at early stages and prior to the development of PD symptoms. By contrast, there was a progressive increase from control to PD in the numbers of CD68-positive microglia/macrophages, a marker associated with phagocytosis, although an increase in the number of microglia was not identified [89]. Walker et al. examined the differential expression of inflammatory and trophic molecules in the SNc and striatum of control, iLBD and PD cases and found distinct patterns of inflammation and growth factor changes [91], which was also reinforced by animal studies [121]. Another piece of evidence suggesting early immunological changes came from the work of Galioano-Landeira et al*.* The authors found that CD8-positive T-lymphocytes were increased in the SNc of PD cases compared to the control group, whereas CD4-positive T cells remained unchanged [92]. Most notably, a robust infiltration of CD8-positive T-cells has been observed prior to the appearance of LBP (Braak Stage 1) and in the absence of DA cell death. CD8-positive T-cells were found to be equipped with cytolytic enzymes (granzymes A, B and K) and proinflammatory cytokines (interferon gamma) with phenotypic differences between early and late stages. A high proportion of nigral CD8 T cells were identified as tissue-resident memory T cells. These results identified a substantial nigral cytotoxic CD8-T-cell infiltration as an early pathogenic event preceding LBP and DA cell death in PD. This further highlights microenvironmental changes which may impact later nigral cell survival. In another study by Hurley et al., iLBD cases had an increased number of IBA1-positive microglia. In the anterior cingulate cortex (ACC), PAR2-positive microglia were increased in iLBD, while in the primary motor cortex, tyrosin-1 was increased in microglia. However, TH-positive neurons in the SNc only showed a decreasing trend [90]. Doorn et al. investigated microglia activity by quantifying the minichromosome maintenance protein 2 (MCM2), a cell proliferation marker. The authors found MCM2-positive cells to be increased in the hippocampus (HC) of iLBD cases but not in established PD patients. This study thus suggests an early microglial response in the HC, indicating that neuroinflammatory processes play an essential role in developing PD pathology [88]. Finally, in another study, the tissue from different Braak stages was examined for the presence of integrin α_v_β_3_, a marker for angiogenesis, along with vessel number and activated microglia. In this study, all PD cases had greater levels of α_v_β_3_ in the SNc compared to controls. PD subjects also had increases in microglia number and activation in the SNc, suggesting a link between inflammation and clinical disease, whereas microglia activation in iLBD subjects was limited to the LC, an area involved in early-stage PD [89]. In summary, immune-associated changes appear to occur early during disease progression, and, consequently, anti-inflammatory strategies may be potentially disease-modifying for PD. Indeed, several anti-inflammatory drugs have been tested for their therapeutic potential in PD. For instance, statins have been proposed to exert neuroprotective effects in PD models through an anti-inflammatory response, improving motor function and attenuating the increase in inflammatory cytokines. Simvastatin, for example, effectively crosses the blood–brain barrier and is currently being studied in a phase 2 randomized, placebo-controlled futility trial [122]. Although recently announced results indicated futility for slowing the progression of PD, an anti-inflammatory approach may require early treatment before LBP-related cell death to yield successful therapeutic effects [123–125]. Other clinical trials investigating anti-inflammatory agents are also still ongoing [126, 127].

### Early synaptic pathology in LBD

Mounting evidence indicates that SNc DA neuron degeneration is likely to start from synaptic pathology [128] and that the loss of synaptic connectivity may precede nerve cell loss. As early as 1989, by analysing vesicular monoamine transporter 2 (VMAT2) binding during ageing in PD and healthy subjects, Sherman et al. provided the first evidence indicating that PD symptoms appear when the striatal denervation state is over a critical threshold of about 50% [82, 93, 94]. This illustrated the relevance of synaptic terminal degeneration in the onset of disease and its clinical phenotype [129]. Schulz-Schaeffer et al. reported that αSyn pathology mainly involves synaptic compartments and proposed that the first neuronal compartment affected by its deposition might be the synaptic terminal [95]. In accord with an early synaptic pathology in PD, most αSyn aggregates accumulated at presynaptic terminals in paraffin-embedded tissue blots from LBP cases [130]. Thus, at the onset of clinical motor symptoms, the loss of DA synaptic terminals exceeds the loss of DA cell bodies, pointing towards an early alteration of synaptic projections that precede neuronal death.

Moreover, neuroanatomical studies of post mortem brain samples from familial PD cases support the idea that synaptic decay precedes neuronal death [131, 132]. These observations support a ‘dying back’ hypothesis where synaptic demise, including presynaptic dysfunction, occurs prior to neuronal death [133, 134]. This view is supported by a series of preclinical studies indicating that αSyn aggregation at synaptic sites impairs neuronal function and axonal transport by affecting synaptic vesicle release [135]. Numerous studies found pre- and postsynaptic structural integrity alterations in PD and Dementia with Lewy bodies (DLB) [46, 130, 136–139]. Furthermore, apart from αSyn, several other PD-associated proteins such as leucine-rich repeat kinase 2 (LRRK2), parkin, DJ-1, PINK1, Rab38B and synaptojanin have been found to be involved in the control of DA synaptic function [140–145]. In accord with an early synaptic dysfunction in PD, various in vivo imaging studies demonstrated presynaptic neurotransmitter deficiencies in PD [136]. These findings seem to indicate that the degenerative process in PD is – at least in part – located at the presynapse, ultimately resulting in a neurotransmitter deficiency syndrome [146, 147]. This degeneration of synapses appears to emerge before motor symptom onset; however, the exact timeline of this progression and its clinical correlates are yet to be fully elucidated. Another critical aspect of these studies is that none of such results were derived directly from iLBD cases, and, although it is conceivable that, for instance, oligomeric non-aggregated αSyn species affect synaptic function prior to the appearance of typical LBs, the specific significance of such αSyn species remains uncertain.

### Changes in gene expression & cell types

A relevant study on early transcriptomic changes in PD was conducted by Wilma van den Berg's group using RNA microarrays [96]. The authors aimed to elucidate molecular mechanisms underlying neuronal dysfunction and LBP in the pre-motor phase of PD and investigated the transcriptome of the SNc of well-characterised iLBD, PD and age-matched controls. Before SNc-LBP, at Braak stages 1-2, they observed deregulation of pathways linked to axonal degeneration, immune response, and endocytosis, including axonal guidance signalling, mTOR signalling, eIF2 signalling and clathrin-mediated endocytosis in the SNc. The results indicate molecular mechanisms related to axonal dysfunction, endocytosis and immune response are already affected before LBP reaches the SNc, while mTOR and eIF2 signalling is also impaired during later stages.

Interesting work implicating additional cell types in iLBD came from a study that integrated genome-wide association study results with single-cell transcriptomic data from the entire mouse nervous system to systematically identify cell types underlying brain complex traits [98]. When applying expression-weighted cell-type enrichment (EWCE) to data from previous studies [148, 149], the authors found that downregulated genes in PD were enriched explicitly in DA neurons (consistent with the loss of this particular cell type in the disease). In contrast, upregulated genes were significantly enriched in cells from the oligodendrocyte lineage. When analysing gene expression data from post mortem human brains, downregulated genes were not enriched in DA neurons at Braak stage 1–2. Conversely, upregulated genes were already strongly enriched in oligodendrocytes at this stage, thus indicating that their involvement precedes the emergence of pathological changes in the SNc. In summary, this study thus supports an early alteration of oligodendrocytes preceding LBP in PD, although the data were in part based on investigating mice.

This finding was corroborated by a recent single-cell study where significant associations were found between reported PD risk genes and highly expressed genes in oligodendrocytes. Furthermore, the risk for PD age of onset was associated with genes highly expressed in oligodendrocyte precursor cells [99]. These studies thus support an early alteration of oligodendrocytes and their precursors, preceding LBP in PD. A study by Santpere et al. investigated global transcriptional changes in the frontal cortex (Area 8) in iLBD, PD and DLB. The authors identified different co-expressed gene sets associated with disease stages. They conducted a functional annotation of iLBD-associated modules using the gene ontology framework categories enriched in gene modules and differentially expressed genes, including modules or gene clusters correlated to iLBD. These clusters revealed upregulated dynein genes and taste receptors and downregulated genes related to innate inflammation [100], thus demonstrating transcriptomic alterations in cortical brain areas in iLBD. In 2012, a study by Lin et al. investigated the extent of mtDNA mutations in early-stage PD and iLBD cases and found that mtDNA mutation levels in SNc neurons are significantly elevated in these cases [97]. However, this study defined iLBD by the absence of clinical parkinsonism or dementia but with Lewy bodies present in the SN, which corresponds to Braak stage 3. These findings illustrate the widespread transcriptomic changes preceding LBP, affecting various cell types, and deregulating crucial molecular pathways.

### Proteomic changes

Changes in the expression of various additional proteins have also been demonstrated, for instance, by Wilhelmus et al., who reported an aberrant ApoE and low-density lipoprotein receptor-related protein 1 expression in SNc DA neurons in PD and iLBD cases. The authors concluded that alterations in lipoprotein homeostasis/signalling in DA neurons of the SNc constitute an early disease event during PD pathogenesis [101]. Likewise, changes in neuropeptides and glutathione levels were found in iLBD [102, 103]. Wilkinson identified changes in the glycosylation of proteins in iLBD: a total of 70 O-glycans were identified, with iLBD exhibiting significantly decreased levels of mannose-core and glucuronylated structures in the striatum and PD presenting an increase in sialylation and a decrease in sulfation [104]. Early oxidative damage in the frontal cortex of iLBD cases has been suggested by a study that investigated lipoxidation of the glycolysis-associated enzymes aldolase A, enolase 1, and glyceraldehyde dehydrogenase (GAPDH) [150] and likewise early work from Jenner et al. suggested a loss of glutathione (GSH) to be associated with iLBD [103, 105, 106]. These proteomic modifications furthermore exemplify the various changes in the SNc prior to LBP emergence.

### Changes in neuronal function and excitability in iLBD

Changes in neuronal function and excitability may occur a long time before structural events can be appreciated and recent research began to elucidate the molecular factors governing such early neuronal malfunction. For instance, Tan et al. investigated the effect of αSyn on regulatory molecules in DA SNc neurons and found a loss of the Fragile X Mental Retardation Protein (FMRP) in most neuromelanin-positive neurons of the SNc in human post mortem brain tissue from PD and iLBD cases [107]. Because FMRP regulates the expression and function of numerous neuronal genes [151, 152], these results further suggest that in PD, DA neuron dysfunction is likely to be present long before morphological and histopathological changes and that the loss of FMRP in the SNc may be a key molecular event in these stages (Fig. 2). Loss of FMRP may have beneficial or detrimental effects on neuronal function in the SNc. Tan et al*.* demonstrated that the absence of FMRP ameliorates αSyn-induced DA dysfunction, and suggest that the early loss of FMRP in PD may in fact protective effects in PD. However, as with the aforementioned studies on autophagy, results from investigating αSyn over-expression are difficult to compare with human LBP and its sequential appearance as the specific αSyn species that are present at different time points are not yet known. The specific significance of FMRP for PD disease progression thus remains to be defined.

### Peripheral changes in iLBD

In addition to these reported CNS changes, iLBD cases may exhibit both peripheral and autonomic pathological changes [153, 154]. For instance, a study by Beach et al. examined the presence of LBP in the gut of iLBD, PD and control cases. The authors found that in the vagus nerve, none of the healthy control subjects showed aggregates of phosphorylated αSyn (p-αSyn), while 46% of iLBD and 89% of PD cases were p-αSyn-positive. In the stomach, none of the control subjects had p-αSyn while 17% of iLBD and 81% of PD subjects did [108]. Following these findings, iLBD cases were retrospectively found to exhibit a lower frequency of bowel movements [109]. In a retrospective autopsy-based study of the human submandibular gland, PD and iLBD cases had LBP in the submandibular glands, the cervical superior ganglia, the cervical sympathetic trunk and vagal nerves [110]. Some previous work even suggested the presence of LBP in the spinal cord of iLBD cases [155] and another study, although limited by a small sample size, found a decrease of TH immunoreactivity within epi- and myocardial sympathetic nerve fibres in PD and iLBD [111]. These studies appear to confirm the cumulative results from studying prodromal PD (pPD), where αSyn is present in the peripheral and autonomic nervous system.

## Prodromal PD as a clinical surrogate for early pathological changes

**Fig. 3:**
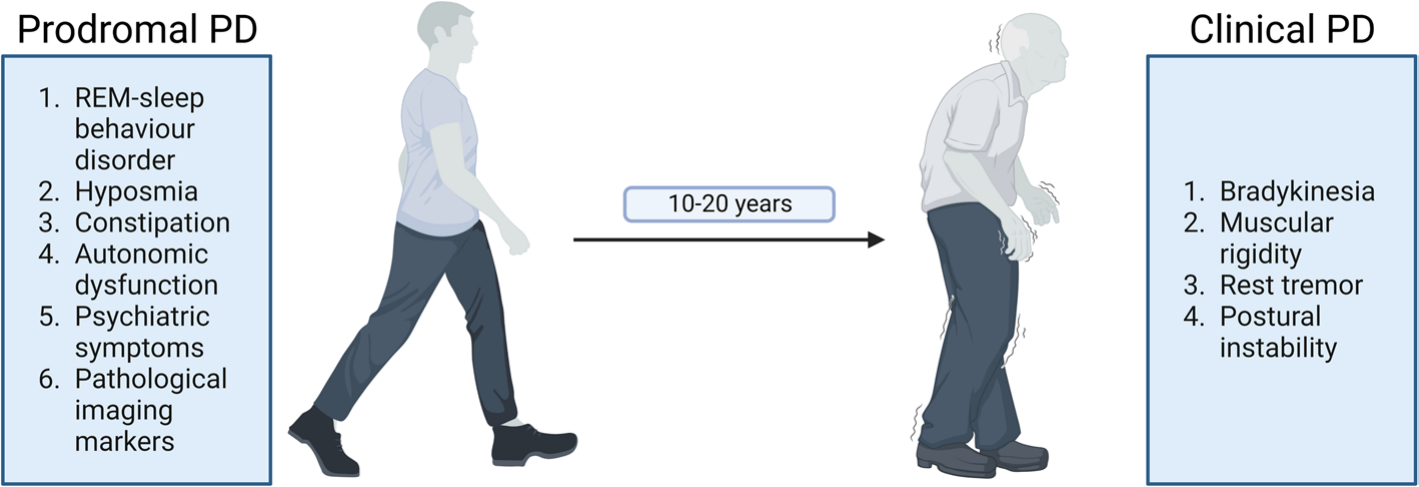
Schematic summarizing clinical signs and symptoms of pPD and the approximate timescale for conversion to Clinical PD. *Created with Biorender.com*

In addition to investigating iLBD, some previous studies investigated cases that exhibit so-called prodromal symptoms: prior to the appearance of the classic motor symptoms during cPD, most PD patients experience several typical non-motor signs that are collectively referred to as pPD (Fig. 3). These signs include REM sleep behaviour disorder (RBD), olfactory loss, constipation, autonomic dysfunction, psychiatric symptoms, and pathological imaging markers of the presynaptic dopaminergic and autonomic nervous system [156, 157]. These prodromal signs and symptoms often precede cPD by 10-20 years [158, 159]. As such, investigating pPD would contribute to understanding early pathological events in PD and indeed, studies that examined pPD have contributed some indirect evidence for early pathological changes, although these results were primarily derived from imaging results. For instance, MRI data from isolated RBD (iRBD) cases showed structural alterations in the SNc and grey matter changes in the motor cortico-subcortical loop correlated with motor abnormalities [160, 161]. iRBD is considered to be an early clinical sign during disease progression with a > 80% risk of conversion to cPD [162–164] within 15 years. Patients typically present with vivid, often frightening dreams that lead to vocalisation and sudden body movements (Fig. 3). In addition to these characteristic sleep disturbances, some iRBD cases may exhibit mild motor deficits [165–168] (Table 2). Such clinical data are consistent with an early affection of extrapyramidal motor areas during disease progression, although the specific molecular correlate remains uncertain. Furthermore, iRBD cases exhibit a reduced striatal dopamine transporter (DaT) binding [169, 170] on [^123^I] Ioflupan scintigraphy and an altered [^18^F]AV133 VMAT2 positron emission tomography (PET) signal [171], further indicating impaired integrity of the nigrostriatal pathway in these cases. Reduced DaT binding also seems to be correlated with changes in brain glucose metabolism as assessed by [^18^F] fluorodeoxyglucose ([^18^F]FDG) PET [172]. Likewise, iRBD cases exhibit impaired nigrostriatal connectivity as assessed by fMRI and ultrasound [161, 173–183] (rev. in [184]). In accord with the aforementioned pathological studies in iLBD, a study examining inflammatory changes in the SNc by [^11^C]PK11195 18 kDa translocator protein (TSPO) PET found increased microglial activation in iRBD, suggesting early immunological changes in the midbrain [120]. Furthermore, Imidazoline 2 imaging with [^11^C]BU99008 PET indicated activated astrocytes in early PD but even decreased tracer signal at late stages compared to healthy controls [185]. These imaging results thus collectively confirm an early and possibly inflammatory pathology in the PD midbrain. Overall, these observations thus indicate that in iRBD, the disease process extends beyond the sleep-related structures in the brainstem to other structures, including the nigrostriatal system [186, 187]. As a limitation of considering iRBD as pre-LBD cases, it is noteworthy that iRBD cases may exhibit LBP at different Braak stages, including those > 3, as substantiated by clinical findings [188] and that in some cases, iRBD may develop into Multiple System Atrophy (MSA) or Dementia with Lewy bodies (DLB) instead of cPD [164, 189].

**Table 2:** Table summarizing changes in the midbrain of pPD cases

Early changes in the pPD midbrain	Ref
**iRBD**	
Mild motor deficits	[165]
↓ Striatal dopamine transporter (DAT) and VMAT2 binding	[169, 170]
Changes in brain glucose metabolism	[172]
↓ Nigrostriatal connectivity	[173–183]
↑ Microglial activation	[120]
↑ Activated astrocytes	[185]
↑ Serum neurofilament light chain (sNfL)	[190]
**Hyposmia**	
↑ Echogenicity of the SNc	[191]
↓ DAT binding	[191]
Correlation between olfactory performance and DAT binding	[192, 193]

In addition to these imaging results, several laboratory results have been derived from iRBD cases. For instance, serum neurofilament light chain (sNfL), a neuronal cytoskeletal protein released upon neuronal damage, might mark the conversion of iRBD to cPD [190]. Techniques such as proteomics analysis of serum samples have identified numerous proteins at significantly altered expression levels, providing further insight into the protein signature profile and molecular pathways involved in the pathogenesis of iRBD [51, 52]. In addition, alterations in circulating microRNAs (miRNAs) have been shown in iRBD. For instance, one study found miR-19b to be significantly down-regulated in iRBD cases that later converted to cPD but not in those who remained disease-free for several years, possibly indicating a role of miR-19b during early disease progression [194]. Still, the diagnostic value of serum miRNA detection remains controversial, as miRNAs show strong pleiotropy. For example, miR-19b has also been implicated in lung cancer progression [195] and schizophrenia [196]. One study revealed decreased antioxidant superoxide dismutase and increased glycolysis in iRBD cases using peripheral blood mononuclear cells [197]. The diagnostic value of other biospecimens, such as those from saliva, tears, or the microbiome, is yet to be explored in patients with iRBD, and longitudinal studies are required to establish whether such biosamples will support the understanding of disease onset and progression in LBP [198]. Finally, novel methods have been developed to investigate αSyn in iRBD cases by using Real-Time Quaking-Induced Conversion assays (RT-QuIC) [199–201]. These assays can detect αSyn seeding activity in different LB-associated conditions with a high sensitivity and specificity [202]. For instance, in a recent study that examined patients with iRBD, RT-QuIC detected misfolded α-Syn in the CSF with both sensitivity and specificity of 90%, and αSyn-positivity was associated with an increased risk of subsequent conversion to cPD or DLB [162]. Along these lines, another report aimed to detect of αSyn aggregates in the olfactory mucosa of a large cohort of subjects with iRBD by RT-QuIC [203]. The authors found the olfactory mucosa to be α-Syn-positive in 44.4% of iRBD cases, in 46.3% of cPD cases, but only in 10.2% of the control subjects. While the sensitivity for iRBD and cPD vs. controls was comparably low (45.2%), the specificity was found to be sufficiently high (89.8%). Compared to immunofluorescent techniques (IF) RT-QuIC was found to exhibit a high diagnostic accuracy [204].

In addition to iRBD, hyposmia is common in cPD (90%) and iRBD (67%) and sometimes precedes motor symptoms by > 20 years (Figs. 2 and 3) [158, 205–207]. The Prospective Validation of Risk Factors for the Development of Parkinson Syndromes (PRIPS) study found that cases of hyposmia had a fourfold risk of converting to cPD compared to normosmic cases [208]. An impaired sense of smell can thus be regarded as an early clinical event during disease progression [209, 210]. However, hyposmia alone is likely a suboptimal predictor for developing cPD since smell loss is relatively common in older adults, and only a minority will develop PD [211]. Concerning pathological changes in midbrain motor circuits, Sommer et al*.* identified 30 patients with idiopathic olfactory loss and found that 11 had increased echogenicity of the SNc on transcranial sonography and 5 cases had impaired DaT binding. This further supports early structural changes during the disease course [191]. Moreover, studies have shown a correlation between olfactory performance and DaT binding in early PD [192]. In another study, 11% of random hyposmic subjects had a DaT deficit at baseline compared to 1% of normosmic subjects [193]. Congruently with clinical studies on olfactory function, a study by Silveira-Moriyama et al*.* examining the *post-mortem* tissue from iLBD, PD and control patients found LBP in all samples from the olfactory bulb and the primary olfactory cortex in iLBD and PD cases [212]. Another study found that in both iLBD and PD tissue, the olfactory bulb was the region most frequently affected by LBP [19]. However, the immediate correlation between hyposmia and LBP in the olfactory bulb has yet to be substantiated since records regarding hyposmia in these patients studied were unavailable.

Collectively, results from investigating pPD further confirm an early structural and functional defect in motor-associated extrapyramidal circuits during PD disease progression that appears to be present prior to the evident appearance of motor signs and symptoms. On the downside, these results provide little conceptual insight into the mechanism of early midbrain neuron dysfunction in PD and no direct correlation with LBP.

## Critical appraisal & future research directions

In the previous sections, we reviewed studies that collectively examined early pathological changes that precede the onset of LBP in the SNc. Investigating these changes has the potential to expose the significance of LBP, reveal early diagnostic and therapeutic targets and ultimately support the development of novel disease-modifying therapies for PD. However, all these approaches have conceptual shortcomings. Although investigating pPD cases by clinical and pathological methods supported the understanding of disease progression on a systemic level and generated valuable predictive data, it provided insufficient insight into the specific molecular and cellular changes occurring prior to LBP and cell death. This limitation applies particularly to areas in the brain stem and midbrain that are difficult to access in detail by routine diagnostics or tissue biopsies, including SNc DA neurons. Similar limitations apply to the neuropathological investigation of genetic PD cases [213], where genetic alterations (*LRRK2, GBA, SNCA*) predict the development of cPD prior to motor symptoms. Second, it is noteworthy that pPD cases may or may not exhibit LBP in SNc neurons, thus confounding the distinction between LBP-dependent and -independent changes. Although some iRBD cases may exhibit mild motor deficits [165–168], indicating SNc dysfunction, it is unclear if this is a consequence of LBP-associated cell degeneration or LBP-independent neuronal malfunction. Thus, investigating pPD does not truly help to clarify LBP's causative role in SNc DA neuron degeneration. Therefore, more research should focus on elucidating the relationship between these individual aspects of early disease events in PD and how they might correlate to one another.

A shortcoming of investigating iLBD relates to the uncertain progression pattern of LBP. Previous work suggested that only about 50% of all PD patients have a distribution of LBP in the brain that is entirely consistent with the Braak staging model, a prerequisite for the assumption that iLBD is a precursor for SNc pathology in PD [29, 214, 215], and about half of PD cases do not seem to show a caudo-rostral spread of LBP throughout the brain [216]. Furthermore, experimental evidence suggested that the spreading of αSyn via autonomic nerve fibres may occur in a caudo-rostral but also rostro-caudal direction [217–219]. In order to explain these distinct spreading patterns in PD, alternative ‘body-first’ and ‘brain-first’ models have been developed [157, 220–222]. As such, a brain-stem LBP would be the most common precursor of cPD, whereas a second route would commence in limbic areas, including the amygdala and progress to the SNc in a rostro-caudal spread. Although these theoretical models may partially explain the experimental inconsistencies, conclusions drawn from iLBD cases may be impeded by the uncertain correlation between clinical and neuropathological progression. Another concern regarding the Braak staging has finally been raised by earlier work form Schulz-Schaeffer et. al. These authors suggested that instead in the form somatic LBs, > 90% of αSyn aggregates are located at the presynapses in the form of very small deposits in PD, while postsynaptic dendritic spines were found to be retracted. Based on these results, the authors hypothesized that instead of LB-associated cell death αSyn aggregate-related synaptic dysfunction may cause neurodegeneration. Although this concept has not been examined in iLBD, it suggests that the traditional neuropathological staging (assessing somatic LBs) may not capture the true onset or progression of LBP, thus limiting its validity [95, 130, 223].

## Conclusion

Here, we summarized cellular and molecular changes occurring in the SNc of iLBD (and pPD) cases. The body of previous work collectively demonstrates numerous pathological changes that appear to precede LBP in PD. These results challenge the current understanding of PD disease progression and the impact of LBP and, in a broader sense, the development of therapeutic strategies that focus on targeting αSyn [224–226]. Therefore, our review may provide a starting point for future studies, which will have to further examin and connect these initial molecular changes occurring in early PD. Our work will support the investigation of novel molecular targets that could halt disease progression before the known neuropathological signs begin to show.

## Data Availability

N.a.

## Abbreviations

PD: Parkinson’s disease
DA: Dopaminergic
SNc: Substantia nigra pars compacta
LBs: Lewy bodies
αSyn: αSynuclein (Protein)
LBP: Lewy body pathology
CNS: Central nervous system
iLBD: Incidental Lewy body disease
DMV: Dorsal motor nucleus of the vagus nerve
AD: Alzheimer’s disease
LRRK2: *Leucine*-*rich repeat kinase 2*
PARK2: Parkinson juvenile disease protein 2
STED: Stimulated emission depletion
PINK1: PTEN-induced kinase 1
cPD: Clinical PD
pPD: Prodromal PD
TH: Tyrosine Hydroxylase
SNARE: Soluble N-ethylmaleimide-sensitive-factor attachment receptor
SNAP29: Synaptosome Associated Protein 29
TLR-2: Toll-like-Rezeptor 2
Cd68: Cluster of Differentiation
ACC: Anterior cingulate cortex
PAR2: Protease activated *receptor* 2
MCM2: Minichromosome maintenance protein 2
HC: Hippocampus
LC: *Locus caeruleus*
VMAT2: Vesicular monoamine transporter 2
DJ-1: Protein deglycase 1
Rab38B: Ras-related in brain
mTOR: Mammalian Target of Rapamycin
eIF2: Elongation initiation factor
mtDNA: Mitochondrial DNA
GAPDH: Glyceraldehyde dehydrogenase
GSH: Glutathion
FMRP: Fragile X mental retardation protein
RBD: REM sleep behaviour disorder
iRBD: Idiopathic RBD
DaT: Dopamine Transporter
PET: Positron emission tomography
TSPO: 18 KDa translocator protein
(pre-)LBD: (Pre-)Lewy body disease
sNfL: Serum neurofilament light chain
DaT: Dopamine Transporter
GBA: Glucocerebrosidase
SNCA: Alpha-Synculein (Gene)
